# Bibliometric and visualized analysis of current advances and future directions in epilepsy: from molecular basis to therapy

**DOI:** 10.3389/fneur.2025.1593621

**Published:** 2025-07-01

**Authors:** Chengqiang Du, Xinlei Xu, Yehui Lv, Fang Tong, Ruofan Lin, Zhifang Yang

**Affiliations:** ^1^School of Basic Medical Sciences, Institute of Wound Prevention and Treatment, Shanghai University of Medicine and Health Sciences, Shanghai, China; ^2^Shanghai First Maternity and Infant Hospital, Shanghai, China; ^3^School of Medicine, Tongji University, Shanghai, China

**Keywords:** epilepsy, ion channel, neurotransmitter, targeted therapy, bibliometrics, visual analysis

## Abstract

**Purpose:**

Through a visual analysis of the literature on epilepsy research in the Web of Science Core Collection, this study aims to explore the molecular basis, providing a reference for scholars and professionals in related fields.

**Methods:**

The search formula is generated using the Mesh keyword list in PubMed. Subsequently, English-language literature is retrieved from the Web of Science Core Collection, with the search period set from January 1, 2015, to December 31, 2024. The study calculates the annual number of publications and citations and analyzes the trends. CiteSpace 6.2. R4 software is used to perform a visual analysis of the retrieved documents.

**Results:**

The search retrieved 1,485 articles related to molecular basis of epilepsy research, with an average annual growth rate of 14.41%. Based on the publication trend line for this period, it is predicted that approximately 208 articles will be published in this field in 2025.

**Conclusion:**

This study examines the dynamic evolution of epilepsy from molecular mechanisms to clinical treatment. Ion channel abnormalities (e.g., KCNQ2 and SCN1A mutations) and neuroinflammatory pathways have become central to basic research, guiding targeted drug design. However, gaps remain between basic research and clinical application, as discoveries like circRNA regulation and glial-neuron interactions have not yet led to effective therapies, and emerging technologies such as optogenetics and nano-drug delivery systems still require clinical validation.

## 1 Introduction

Epilepsy is one of the most common and potentially fatal neurological disorders. Its occurrence is unpredictable, stemming from abnormal neuronal discharge in the brain (1), and it affects approximately 70 million people worldwide. Among every 1,000 adult epilepsy patients, the annual incidence of Sudden Unexplained Death in Epilepsy (SUDEP) is 1.2 (2), and the risk of suicide is 2 to 4 times higher than in the general population (3). This underscores that epilepsy not only impacts physical health but also profoundly affects mental well-being. Thus, investigating the pathogenesis of epilepsy is crucial for early intervention and effective clinical treatment. Meanwhile, the pathogenesis of epilepsy is multifaceted, encompassing genetics, immunology, neurology, and other disciplines. This study focuses on the molecular level, aiming to provide stronger theoretical support for molecular targeted therapies by summarizing ion channels, transporters, and neurotransmitters. The goal is to bridge the gaps between molecular mechanisms, pathological features, and therapeutic strategies, thereby establishing a three-dimensional network that offers valuable insights for clinicians and researchers in related fields.

Currently, research on the molecular mechanisms of epilepsy primarily encompasses three main aspects: (1) the imbalance mechanism of neurotransmitters mediated by ion channels and transporters (4); (2) the dynamic reversal mechanism of excitatory and inhibitory neurotransmitters (5, 6); (3) the exploration of epilepsy models and precise treatment systems (7), including targeted molecular therapies such as gene-targeted therapy (8) and chaperone therapy (9). Additionally, emerging technologies such as optogenetic intervention (10) and artificial intelligence (11) are progressively being applied to clinical trials and treatment. The advancement of omics technologies has also provided new momentum for studying the molecular mechanisms of epilepsy.

**Figure 1:**
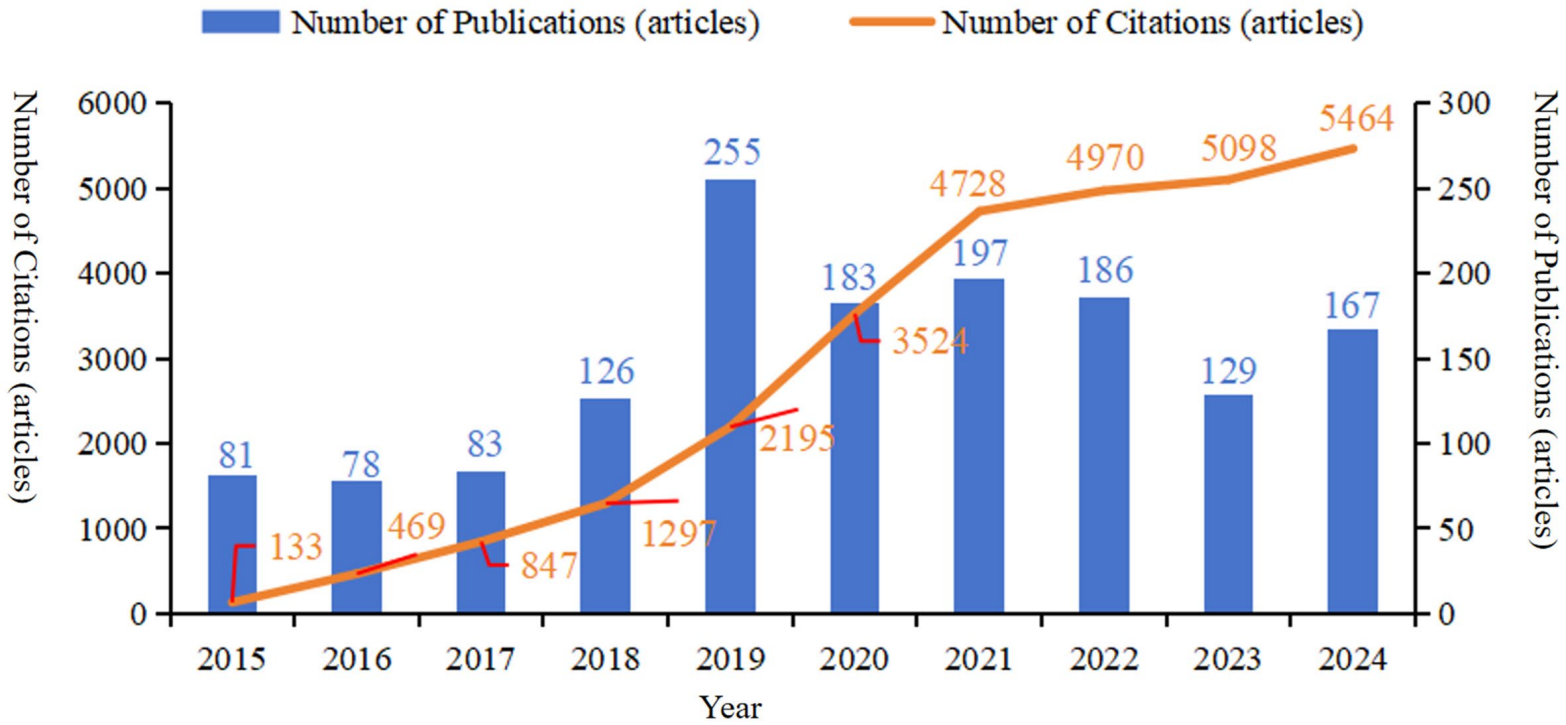
The annual number of publications and citations of literature related to molecular basis of epilepsy research in the core collection of Web of Science database from 2015 to 2024.

Research on the molecular basis of epilepsy holds significant promise for future advancement. Current data indicate (Figure 1) that the number of publications in this field has remained relatively stable, exhibiting only minor fluctuations. However, analysis of the number of citations and publication trends suggests strong potential for continued growth. In this context, bibliometric analysis plays a crucial role in identifying current research hotspots and forecasting future directions in the field. To this end, the present study utilizes CiteSpace software to conduct a visual bibliometric analysis of relevant literature on molecular mechanism of epilepsy, drawn from the core collection of the Web of Science. The objective of this analysis is to provide an intuitive overview of current research hotspots, elucidate the underlying evolutionary trends, and forecast future directions, thereby offering a solid foundation for forward-looking research in this domain.

## 2 Materials and methods

### 2.1 Data source

**Figure 2:**
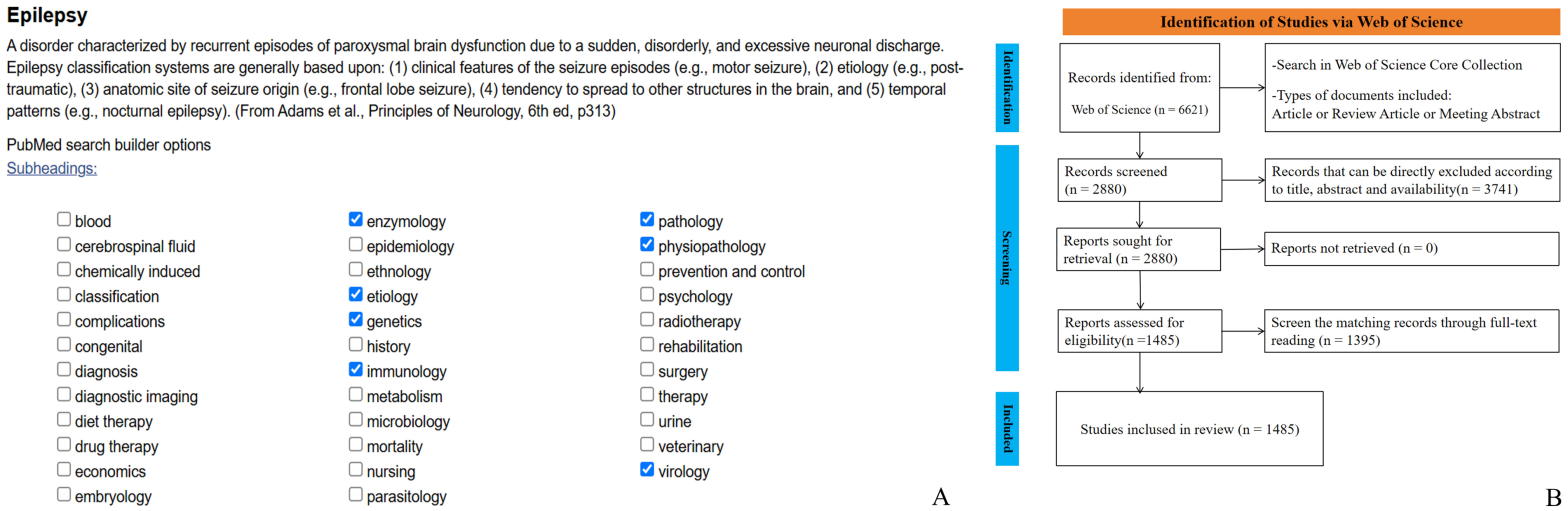
Research process. **(A)** Composition of research formula, **(B)** Search flow chart.

The search formula is generated using the Mesh keyword list in PubMed (Figure 2A). Then, using the Web of Science Core Collection as the data source, a literature search was conducted with the following topic search formula: ((TS = (Epilepsy OR Seizure OR Convulsion OR Epileptic OR Seizure Disorder)) AND WC = (enzymology OR etiology OR genetics OR immunology OR pathology OR physiopathology OR virology)) AND LA = (English). Inclusion criteria comprised articles, review articles and meeting abstracts retrieved from the Web of Science Core Collection using the defined search strategy. Initially, titles and abstracts were screened to exclude publications irrelevant to the study scope outlined in the introduction. Remaining articles were subsequently reviewed in full, as detailed in Figure 2B. Manual screening was conducted independently by one author; in cases of uncertainty, corresponding author was consulted. Disagreements regarding data inclusion were resolved through discussion among all authors. If consensus could not be reached, the data were excluded to ensure the rigor and consistency of the analysis. Throughout the screening process, all reviewers adhered to strict standards to maintain scientific validity and reliability. The search encompasses the entire Web of Science core collection from January 1, 2015, to December 31, 2024. After manual screening, annual publication volume and citation data are calculated, and trends are analyzed.

### 2.2 Visualization analysis items

For the retrieved literature, the annual publication volume and citation counts were calculated, and the trends were analyzed. Microsoft Office Excel 2021 (Microsoft Corporation, United States) was used to create combination charts, and a trend line was used to predict the number of publications in this field for 2025. The annual publication growth rate was calculated using the following formula: Annual Growth Rate (%) = [(Publication volume in the current year − Publication volume in the previous year) / Publication volume in the previous year] × 100.

Meanwhile, the following metrics were analyzed: (1) source journal analysis: the number of journals contributing to the publications was analyzed. Additionally, the top 10 journals by publication volume were examined for their research focus, country of publication, impact factor, citation score, and classification within the Journal Citation Reports (JCR). (2) Citation analysis: the keywords of the cited references were clustered using the Log-likelihood Ratio (LLR) algorithm, and a timeline graph was generated to visually display past and current research hotspots. In the clustering analysis, clusters were ranked by size, with the largest cluster labeled as #0, followed by #1, and so on. (3) Keyword analysis: a keyword clustering analysis was performed, and a timeline graph was generated. Additionally, the burst detection algorithm was used to generate a list of burst keywords (hereafter referred to as “burst terms”), and the top 10 burst terms by intensity were displayed. Burst intensity reflects the frequency change of a burst term over a period of time, with higher intensity indicating more attention. By analyzing high-intensity burst terms, future research hotspots in the field can be predicted.

### 2.3 Data processing

CiteSpace is a bibliometric-based scientific knowledge graph analysis tool used to identify development trends and research frontiers within a specific research field. The literature data is divided into time slices by year, and algorithms such as cosine similarity are applied to generate Keyword Co-occurrence Networks, Co-citation Networks, Collaboration Networks, and other visualizations to display specific relationships. Key metrics such as betweenness centrality, modularity, and mean silhouette score are used to assess the network structure and clustering quality. The resulting figures include co-citation networks, cluster visualizations, and timeline views, reflecting the intellectual foundation and evolution of the field. Keyword co-occurrence maps, clustering diagrams, and burst term timelines highlight research hotspots, thematic structures, and emerging trends.

In this study, CiteSpace 6.2. R4 software was used to perform co-occurrence network and burst analysis on the 1,485 articles. The parameter settings were as follows: time slice was set to 1 year, the scaling factor (k value) was manually adjusted to 15, and the network link strength was calculated using the cosine algorithm. All other options were kept at their default settings. The node types selected for the analysis were journals, countries, cited references, and keywords. CiteSpace uses the Latent Semantic Indexing (LSI) algorithm to extract cluster labels. The software provides two metrics [Modularity Value (Q-value)] and [Mean Silhouette Value (S-value)] to assess the clarity and effectiveness of the visualized clusters. A Q-value greater than 0.3 indicates a significant clustering structure, while an S-value greater than 0.7 suggests that the clustering is highly convincing. An S-value above 0.5 generally indicates that the clustering is reasonable. These metrics help ensure that the visualized network and clusters effectively represent the underlying research patterns.

## 3 Results

### 3.1 Analysis of annual publication volume

**Figure 3:**
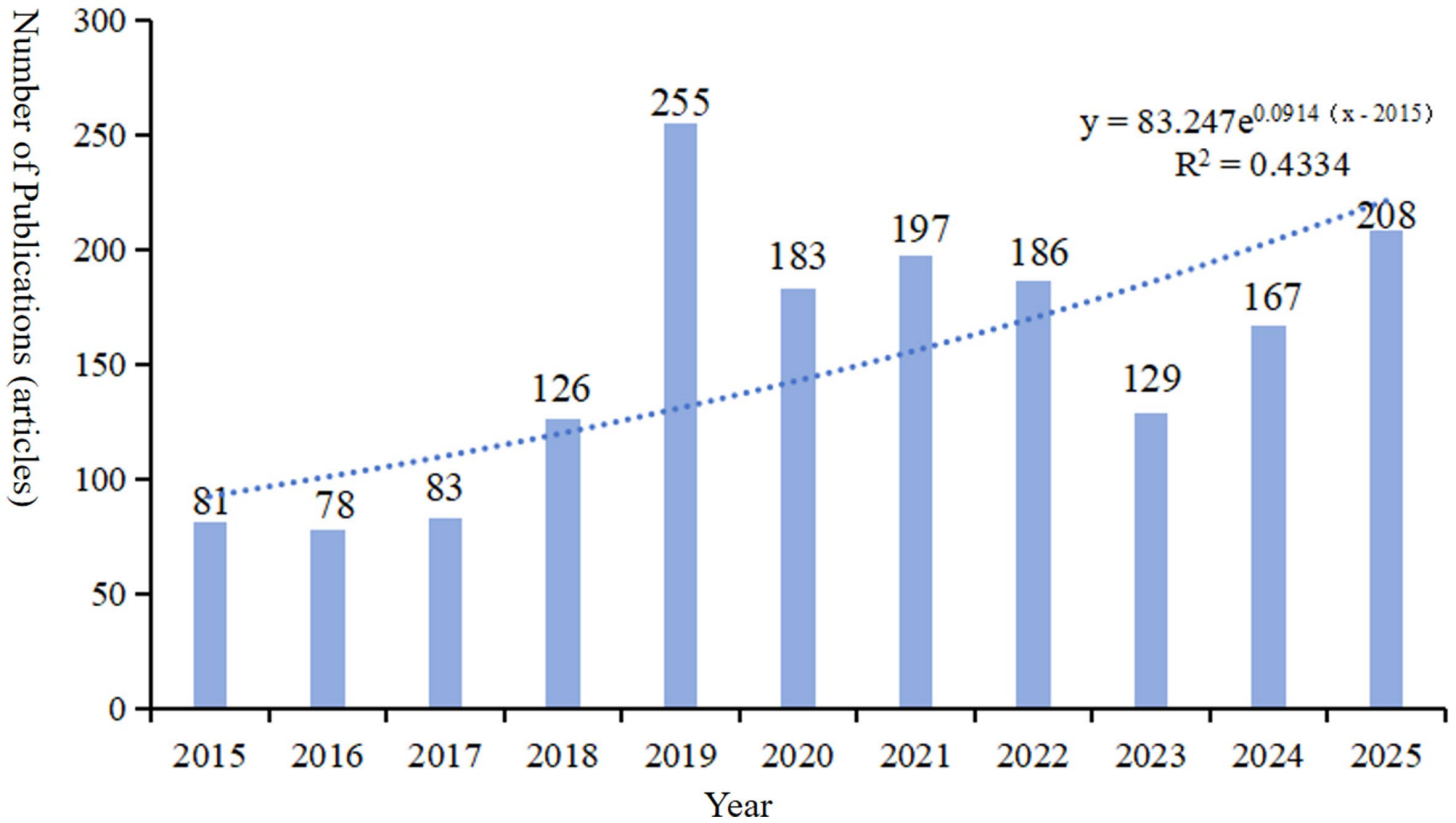
The annual number of publications and trend line of literature related to molecular basis of epilepsy research in the core collection of Web of Science database from 2015 to 2024. The published articles in 2025 is the predicted value of the model.

Based on the searching formula, 6,621 articles were obtained during the pre-detection phase (Figure 2B). To ensure the accuracy and professionalism of the results, the pre-retrieved articles will be manually screened by two researchers, and finally 1,485 articles will be obtained. The final result shows that the research on the molecular basis of epilepsy has demonstrated overall stability, with only slight fluctuations over time. The number of publications peaked in 2019, reaching 255 articles, and the quantity of publications is 2.02 times that of the previous year. Surpassed 100 articles starting in 2018, with subsequent years showing a consistent range around 150 articles. The number of citations has steadily increased year by year, with a significant surge occurring in 2019. By 2024, the number of citations had grown to approximately 50 times that observed in 2015 (see Figure 1). The average annual growth rate of publications from 2015 to 2024 was 14.41%. Based on the trend line for this period (y = 83.247 $e^{0.0914(x-2015)}$ , *R*^2^ = 0.4334; where e is the natural logarithm, x represents the year, and *R*^2^ indicates the goodness of fit, with values closer to 1 representing a better fit), it is predicted that the number of publications in this field will reach 208 in 2025 (see Figure 3).

### 3.2 Analysis of source journals of papers

The searching results identified publications in 207 different journals. Based on publication volume, the top 10 journals were ranked, collectively contributing 516 publications, which accounts for 34.7% (516/1485) of the total. These journals primarily focus on research areas such as Genetics, immunology, pathology, with the majority of publications originating from countries like the United States and the United Kingdom. According to the Journal Citation Reports (JCR) classification standards, four of these journals falls into the first quartile (Q1). See Table 1 for details.

**Table 1:** The top 10 source journals in terms of the number of publications of literature related to the molecular basis of epilepsy research in the core collection of web of science database from 2015 to 2024.

Rank	Journal	Country	Published (Amount)	CiteScore	Journal citation reports (JCR)	IF
1	American Journal of Human Genetics (Am J Hum Genet)	United States	85	14.7	First quartile (Q1)	8.1
2	Human Molecular Genetics (Hum Mol Genet)	England	84	6.9	Second quartile (Q2)	3.1
3	Journal of Neuroinflammation (J Neuroinflamm)	England	58	15.9	First quartile (Q1)	9.3
4	American Journal of Medical Genetics Part A (Am J Med Genet A)	United States	52	3.5	Third quartile (Q3)	1.7
5	Clinical Genetics (Clin Genet)	Denmark	45	6.5	Second quartile (Q2)	2.9
6	European Journal of Human Genetics (Eur J hum Genet)	England	45	9.9	Second quartile (Q2)	3.7
7	Frontiers in Immunology (Front Immunol)	Switzerland	39	9.8	First quartile (Q1)	5.7
8	Genes	Switzerland	37	5.2	Second quartile (Q2)	2.8
9	Brain Pathology (Brain Pathol)	United States	36	13.2	First quartile (Q1)	5.8
10	Frontiers in Genetics (Front Genet)	United States	35	5.5	Second quartile (Q2)	2.8

### 3.3 Analysis of countries (regions) and institutions

**Figure 4:**
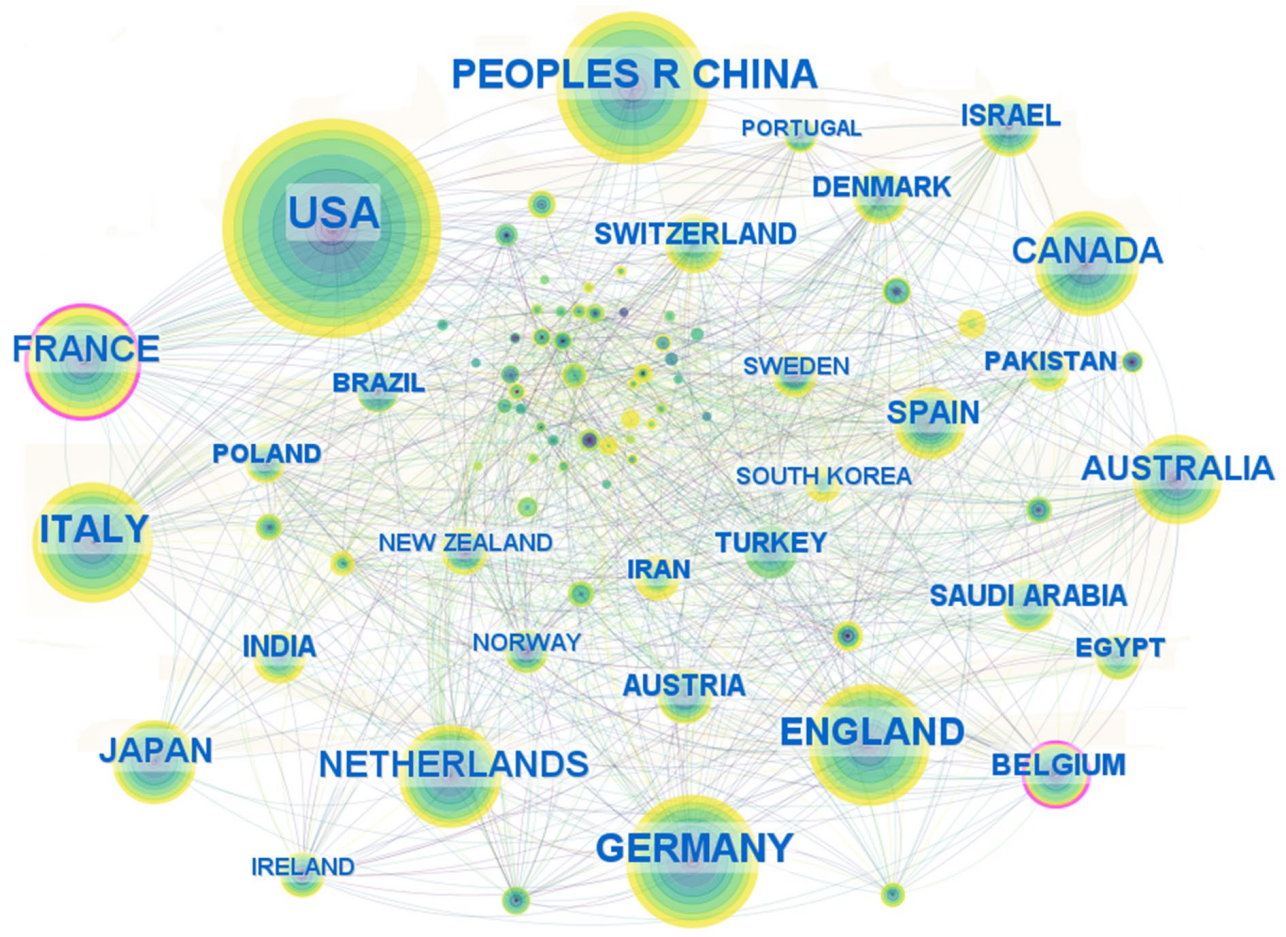
Country (region) collaborative networks for research field in molecular basis of epilepsy.

A collaboration network map of countries (regions) and institutions was constructed based on the findings from the literature search. As depicted in Figure 4, the map illustrates the cooperative relationships among various countries, with the thickness of the connections indicating the intensity of cooperation. A thicker connection signifies a closer and stronger collaborative relationship. The United States, with 566 publications, represents the largest node, accounting for 38.11% of the total literature, and ranks first in terms of publication volume. The People’s Republic of China ranked second with 292 publications (19.7%), followed by Germany in third place with 223 publications (15.0%). Additionally, France exhibits the highest betweenness centrality, scoring 0.13. Other countries (regions) with betweenness centrality scores exceeding 0.1 include Belgium (0.11). Germany, the Netherlands, Norway, and Saudi Arabia are tied for fourth place with a betweenness centrality of 0.08.

**Figure 5:**
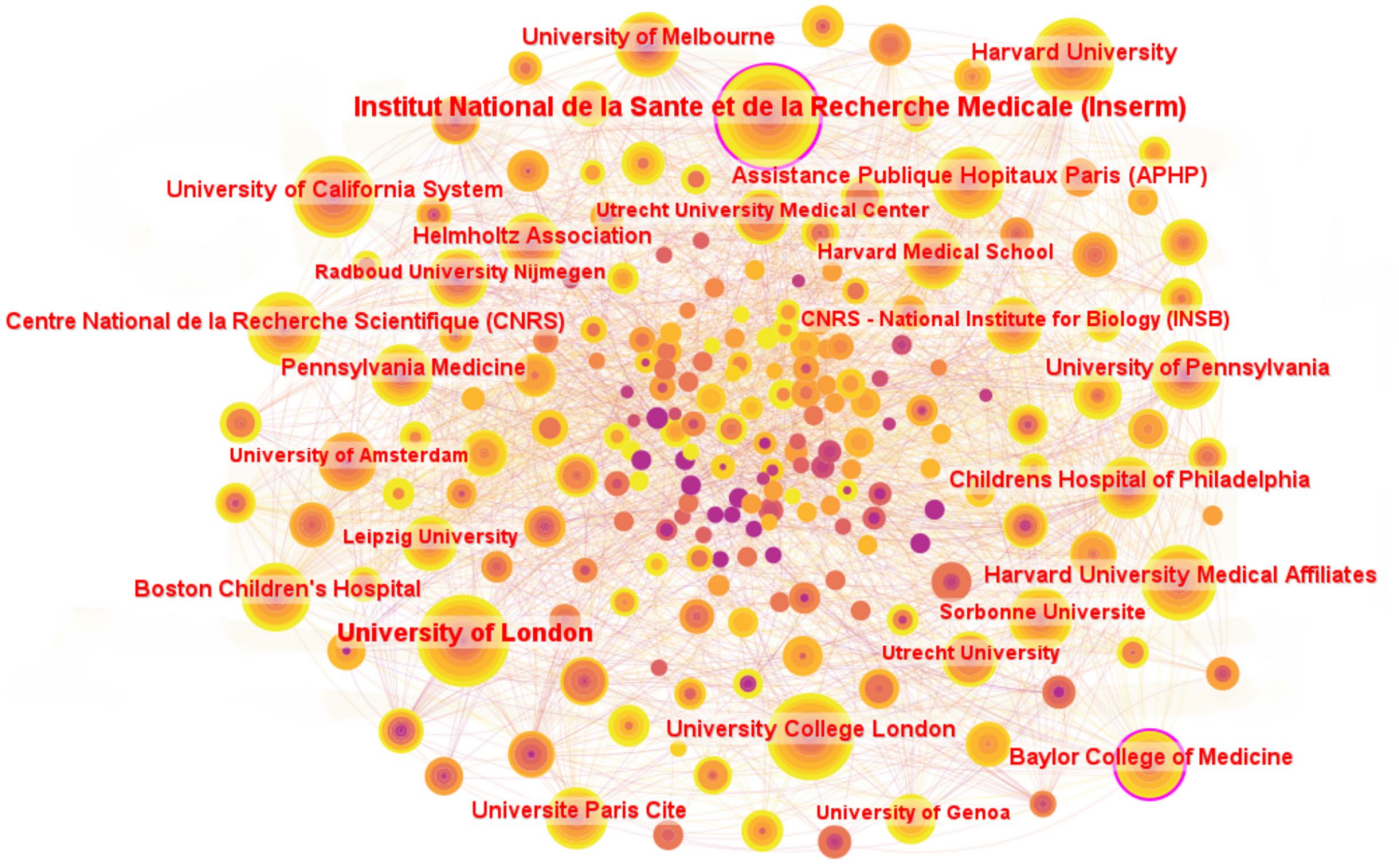
Institutions collaborative networks for research field in molecular basis of epilepsy. Only institutions with ≥27 publications are included in the figure.

A total of 247 institutions have contributed to research on the molecular basis of epilepsy. Figure 5 presents the top 25 institutions ranked by publication count. Hôpital Universitaire Pitié-Salpêtrière-APHP and Leipzig University share the 25th position, each contributing 27 publications. Among the top five institutions by publication volume, Institut national de la santé et de la recherche médicale (Inserm) leads with 121 papers, followed by the University of London (96 papers), Harvard University (89 papers), University College London (86 papers), and the University of California System (82 papers).

### 3.4 Keyword analysis

**Figure 6:**
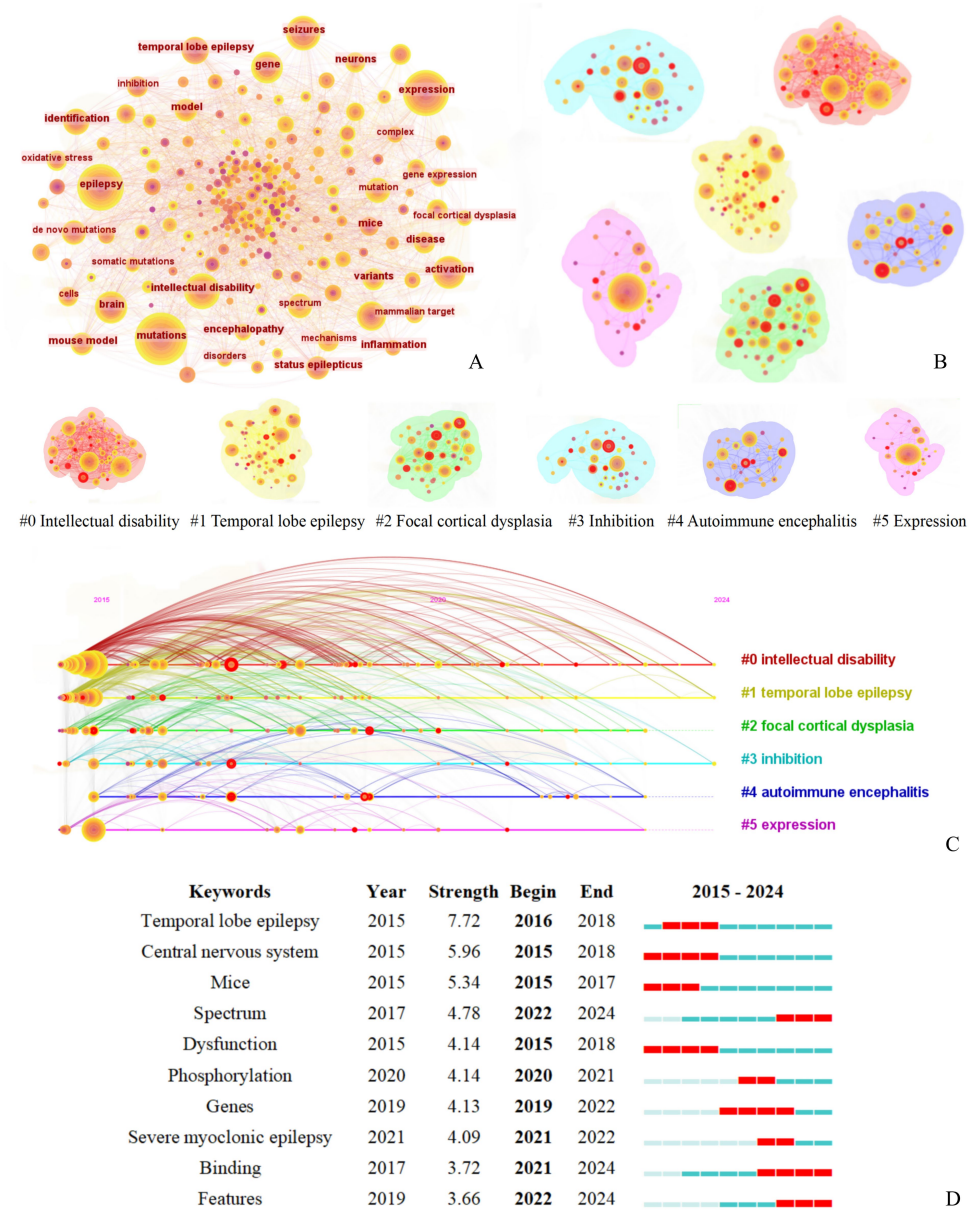
Keyword co-occurrence cluster analysis of literature related to molecular basis of epilepsy research in the core collection of Web of Science database from 2015 to 2024. **(A)** Keyword Co-occurrence Network figure, **(B)** Keyword Co-occurrence Analysis Cluster Network figure, **(C)** Timeline of the Top Six Clusters in Keyword Co-occurrence Analysis, **(D)** Top 10 Burst Keywords by Burst Intensity [The legend corresponds to figure part label **(B)**].

A comprehensive analysis was conducted on the keywords from the literature search, and the keyword co-occurrence knowledge map is shown in Figure 6A. The map consists of 198 nodes and 1,566 lines. Among them, “activation” has the highest betweenness centrality at 0.13, ranking first, followed by “expression” at 0.11. “identification” and “epilepsy” all rank third with a centrality of 0.09. As shown in Figure 6A and the gene-related terms in Table 2, the term “expression” primarily reflects research at the genetic level, particularly involving gene expression and frequent gene mutations. In fields such as immunology, the pathogenesis of epilepsy is often explored through the role of stage-specific gene expression (12). In contrast, the term “activation,” which holds the highest centrality, appears to span multiple biological processes, including the activation of diverse cellular signaling pathways, such as those involving protein C receptors (13) and AAV vectors (14).

**Table 2:** Top 10 most cited publications on the molecular basis of epilepsy (2015–2024) in the web of science core collection.

Rank	Count	Title	Centrality	Sigma
1	76	Analysis of protein-coding genetic variation in 60,706 humans	0.23	4.96
2	53	The mutational constraint spectrum quantified from variation in 141,456 humans	0.19	19.86
3	52	Standards and guidelines for the interpretation of sequence variants	0.11	3.55
4	40	De novo mutations in epileptic encephalopathies	0.27	35.98
5	35	GeneMatcher: a matching tool for connecting investigators with an interest in the same gene	0.02	1.17
6	33	ILAE classification of the epilepsies: Position paper of the ILAE Commission for Classification and Terminology	0.41	12.45
6	33	Prevalence and architecture of de novo mutations in developmental disorders	0.19	1.70
7	24	A general framework for estimating the relative pathogenicity of human genetic variants	0.04	1.23
8	19	CADD: predicting the deleteriousness of variants throughout the human genome	0.06	1.30
9	18	VarSome: the human genomic variant search engine	0.05	1.32
9	18	Epilepsy in adults	0.04	1.36
10	16	Neuroinflammatory pathways as treatment targets and biomarkers in epilepsy	0.30	3.72

Collectively, these findings suggest that research on the molecular basis of epilepsy follows a relatively structured approach: specific pathways are activated and gene expression patterns are analyzed to elucidate disease mechanisms and ultimately inform treatment strategies (14). This reflects the multi-dimensional network that the present study aims to reveal.

The interactions between keywords were analyzed using LSI to extract clustering labels, resulting in an average silhouette value of S = 0.67, indicating that the clustering structure is significant and the results are convincing. Six main clusters were formed: #0 Intellectual disability, #1 Temporal lobe epilepsy, #2 Focal cortical dysplasia, #3 Inhibition, #4 Autoimmune encephalitis, #5 Expression, as shown in Figure 6B.

Burst word analysis identified 47 burst terms, with the top 10 burst terms (Figure 6C) being temporal lobe epilepsy, central nervous system, mice, spectrum, dysfunction, phosphorylation, genes, severe myoclonic epilepsy, binding, and features, each with different starting and ending timeframes. In the early to mid-period (2015–2020), the construction of various experimental mouse models remains a prominent approach for exploring pathogenesis, with temporal lobe epilepsy being a particularly focal disease of interest. Since 2020, the pathological manifestations were linked to the underlying pathogenesis and were analyzed comprehensively from multiple dimensions.

### 3.5 Citation analysis

**Figure 7:**
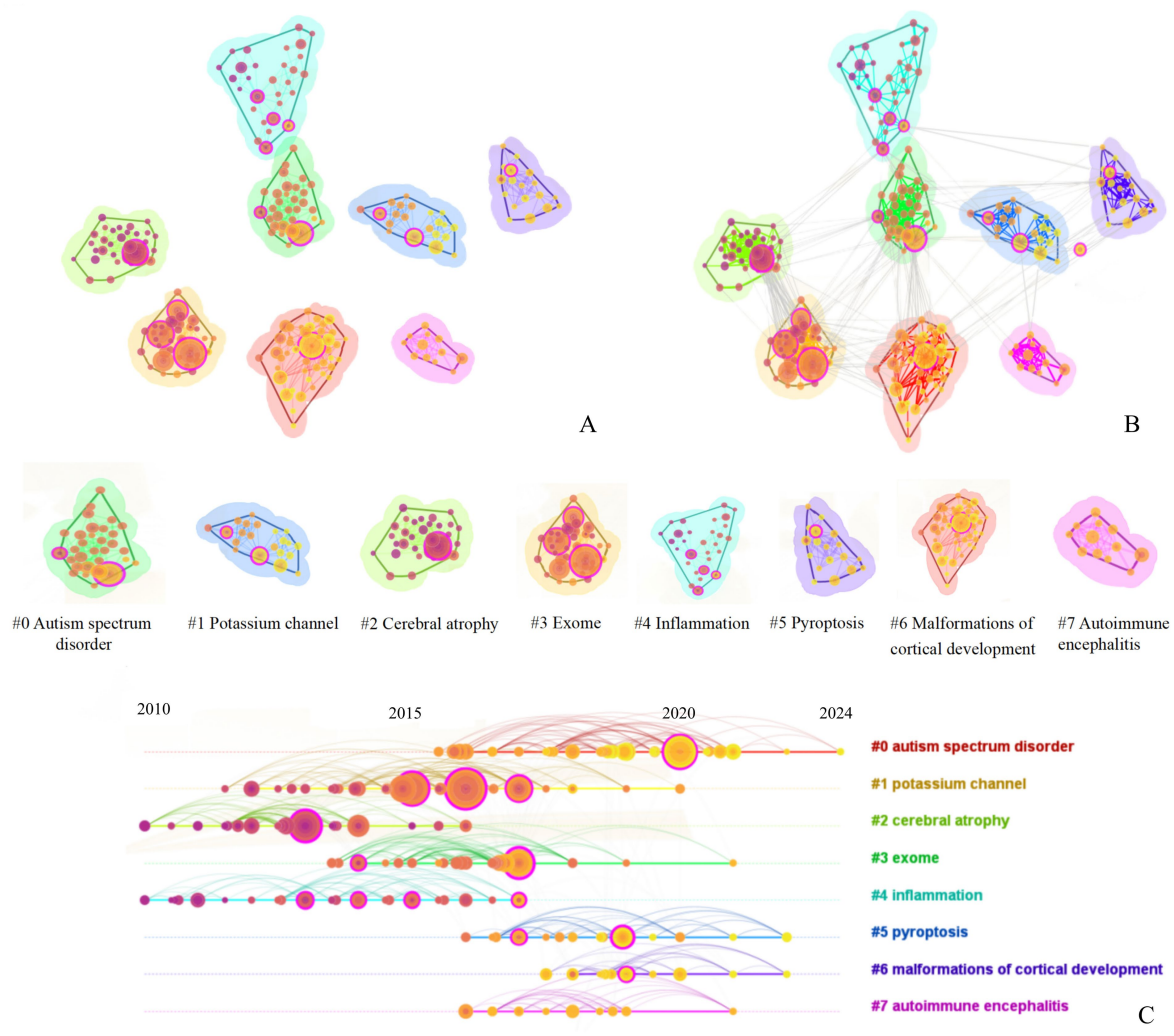
Keyword cluster analysis of cited literature of literature related to molecular basis of epilepsy research in the core collection of Web of Science database from 2015 to 2024 [The legend corresponds to the figure part labels **(A,B)**].

Cluster analysis revealed that the keywords of the cited references formed 253 nodes and 8 major clusters (Q = 0.68, S = 0.87), as shown in Figure 7A. The timeline chart indicates that the primary clusters from 2010 to 2015 were #2 cerebral atrophy, #4 inflammation, while the primary clusters from 2016 to 2020 were #0 autism spectrum disorder, #1 potassium channel, #2 Putrefaction, #3 exome, as shown in Figure 7C. Among these, #2 cerebral atrophy and #3 exome showed the closest co-occurrence relationship, followed by #0 autism spectrum disorder and #6 malformations of cortical development, as seen in Figure 7B.

According to the number of citations, the top 10 literatures were ranked and their betweenness centrality and sigma value were analyzed. As shown in Table 2, research on the molecular basis of epilepsy is largely concentrated at the genetic level. The most cited article, “*Analysis of protein-coding genetic* var*iation in 60,706 humans*,” provides a comprehensive map of protein-coding variants, serving as a key resource for identifying epilepsy-related mutations. The second and third most cited works offer standardized frameworks for variant interpretation, further supporting the identification of pathogenic mutations. The sigma value = (centrality + 1)^burstness. Consequently, higher centrality and salience values correlate with a higher sigma value. In terms of centrality, the second-ranked publication, “*Neuroepidemiological pathways as treatment targets and biomarkers in epilepsy*” (centrality = 0.30), reflects a growing focus on inflammatory mechanisms and their therapeutic potential. This also highlights the interdisciplinary nature of the field, where multi-dimensional and multi-level analyses contribute to more comprehensive research outcomes.

Overall, these influential studies form the foundation of molecular epilepsy research, spanning genetic data construction, pathogenic mechanism discovery, variant assessment tools, and clinical classification systems—demonstrating a shift toward more systematic and precise investigation in this field.

## 4 Discussion

Bibliometric analysis is a type of study that statistically analyzes scientific articles to describe citation relationships across publications and research trends in a certain field, being valuable for statistically evaluating researcher contributions across countries (15). In the present study, we performed bibliometric analysis to understand worldwide trends in postmortem interval research, focusing on specific topics within the discipline across a nearly 10-year period. To the best of our knowledge, this is a study to perform bibliometric analysis on the global scientific body of research on postmortem interval via the Web of Science Core Collection databases.

The number of scientific publications is an intuitive indicator that could reflect the development of the discipline based on bibliometrics (16). The substantial variation in the number of articles published annually may signal a significant turning point in this field. Therefore, by integrating the timeline and publication trend charts with the keyword co-occurrence map, we can gain a clearer understanding of the current molecular mechanisms underlying epilepsy, identify research hotspots, and anticipate future research directions. 2019 marked the peak in the number of articles published over the past decade. Following a breakthrough of over 100 articles in 2018, the number of publications peaked at 255 in 2019, with annual fluctuations between 120 and 180 and an average of approximately 150 articles per year, suggesting that the molecular basis for studying the pathogenesis of epilepsy has reached a relatively mature stage. The number of citations has steadily increased each year, with an exponential rise in 2019, indicating that epilepsy research spans a broad range of fields and continues to hold significant value for further investigation.

As per the top 10 journals list, American Journal of Human Genetics, Human Molecular Genetics, Journal of Neuroinflammation and American Journal of Medical Genetics Part A made the most contributions to scientific research, and the number of published articles has exceeded 50. Only one of the top 10 journals is classified in Q3, while the remaining journals are ranked in Q1 and Q2. This distribution further highlights the professionalism and rigor of research in this field. Similarly, the keyword analysis further demonstrates that research on the molecular mechanisms of epilepsy follows a relatively structured framework. Insights into molecular mechanisms ultimately inform the understanding of epilepsy severity and provide guidance for clinical treatment strategies. For instance, the high-centrality keyword “activation” encompasses multiple aspects of epilepsy molecular research. Activation of neuroinflammatory pathways represents a key approach to exploring epileptogenic molecular mechanisms as exemplified by the article “*Neuroinflammatory pathways as treatment targets and biomarkers in epilepsy*,” which ranks second in centrality in Table 2. Furthermore, an analysis of the cited literature reveals that most studies focus on genetic approaches, with many explaining the molecular basis of epilepsy by investigating mutation sites. Simultaneously, attention is also given to the emergence of specific epilepsy categories, which contributes to the development of a more comprehensive, three-dimensional knowledge network encompassing the “molecular mechanisms, pathological characteristics, and treatment strategies” of epilepsy. As for research hotspots and trends, a more detailed explanation is provided in the following text.

### 4.1 Current research status and hotspot analysis of epilepsy in molecular basis

The intensity (Figure 6B) and high dominance (Figure 6D) of temporal lobe epilepsy are evident from the timeline diagram, keywords, and their emergent maps. Temporal lobe epilepsy is the most common form of focal epilepsy in adults, accounting for 60% of cases (17). Additionally, it is a common practice in research to construct mouse models of temporal lobe epilepsy in laboratory settings (18), which explains the prominence of keywords such as “mouse,” “expression,” and “model.” Current research primarily focuses on the imbalance mechanisms of neurotransmitters mediated by ion channels and transporters, such as mutations in sodium channels (19), abnormal regulation of potassium channels (20), dynamic imbalances in calcium channels (21), and new discoveries related to transporters (22). The International League against Epilepsy (ILAE) classifies epilepsy caused by ion channel gene mutations as “genetic epilepsy” and underscores its significance in the classification and diagnosis of epilepsy (23). This also underscores the prominent role of ion channel research in elucidating the molecular basis of epilepsy. As shown in Table 2, the articles published by the International League against Epilepsy have the highest central value in the cited literature.

Voltage-gated sodium (Nav) channels play a crucial role in the depolarization of action potentials, thereby significantly influencing nerve conduction. In the brain, the voltage-gated sodium channel family (SCN) primarily encodes Nav channel proteins. The SCN2A, SCN3A, and SCN8A genes encode three major *α* subunits, which are predominantly expressed in excitatory neurons (24). In contrast, SCN1A is highly expressed in inhibitory interneurons. However, due to the high mutation rates and varying expression patterns of these genes, there is an imbalance in the excitation/inhibition ratio, which leads to diverse seizure patterns and syndromes. Mutations such as truncations or missense mutations in the SCN1A gene, which encodes the αsubunit of the Nav1.1 channel, can lead to Dravet syndrome (25), SCN1A mutations are frequently associated with a spectrum of clinical manifestations, including epilepsy, autism, and intellectual disability. The emergence of high-intensity keywords like “Dysfunction” and “Severe myoclonic epilepsy” in Figure 7 is closely associated with this, highlighting the pathological characteristics of epilepsy. Current studies indicate (26) that the spectra of phenotypes and genotypes of SCN1A mutations have been expanding, then the incidence and severity of associated clinical symptoms may vary accordingly. Meanwhile, intellectual disability in cluster #0 occupies the largest share in keyword clustering, which is consistent with the finding that epilepsy, autism spectrum disorder, and intellectual disabilities are highly correlated in this research area (27). This study aims to more thoroughly investigate the molecular mechanisms underlying epilepsy and to establish a comprehensive network integrating molecular mechanisms, pathological features, and therapeutic strategies through multidisciplinary and multi-angle approaches. Currently, treatment options for SCN1A-related disorders have become more mature and diversified. One approach involves constructing an AAV-SCN1A vector, which increases SCN1A mRNA transcripts, particularly in GABA-inhibited intermediate neurons, as well as the level of Nav1.1 protein in the brain (19). Additionally, the development of cutting-edge models, such as using Scn1a haploid-deficient mice for gene therapy based on CRISPR, and fusing multiple guide RNAs (gRNAs) in the promoter region with nuclease-deficient Cas9 and a transcription activator (dCas9-VPR) to trigger SCN1A transcription *in vitro*, has opened up new therapeutic avenues (28). With advancements in science and technology, precision therapy has also paved the way for novel treatments. For instance, gene targeting therapy using SCN1A antisense oligonucleotide (STK-001) can restore Nav1.1 expression (29), while chemical chaperone 4-PBA can correct the folding of SCN1A missense mutant proteins through chaperone protein therapy (30). Gene targeting and chaperone protein therapies are emerging as prominent topics in precision medicine, and it is expected that further research will lead to new breakthroughs in these areas.

Additionally, cluster #2, which focuses on focal cortical dysplasia (FCD), also shows a high incidence, as not only mutations in SCN1A and SCN2A that lead to excessive neuronal excitation result in FCD (31, 32), but mutations in genes encoding potassium channels can also produce similar outcomes (33). Voltage-gated potassium channels (Kv) play a crucial role in regulating electrical excitability within the neuronal system, similar to the function of sodium channels. Mutations in Kv channels have been linked to epilepsy. As demonstrated by case reports and studies utilizing knockout mouse models, both gain-of-function (GOF) and loss-of-function (LOF) mutations in Kv channels can lead to epilepsy with similar phenotypes, though through different mechanisms, as seen with many other potassium channel families (34). The KCNQ (Kv7) family of voltage-gated potassium channels is associated with various hyperexcitability diseases and GOF variants (35). This family consists of five members (35), with the KCNQ2 channel most commonly linked to developmental epilepsy and epileptic encephalopathy. Potassium channels encoded by KCNQ2 and KCNQ3 regulate subthreshold membrane potential changes that influence neuronal excitability, contributing to the molecular heterogeneity of the M current (36). The M current is a non-inactivating, voltage-dependent potassium current that plays a critical role in modulating neuronal excitability and limiting repetitive neuronal discharges (37). Based on the characteristics of the M-current and specific gene loci, a high-efficiency enhancer has been identified to stabilize the neuronal membrane potential and reduce hyperexcitability. This (38) is achieved by activating KCNQ2/3 (Kv7.2/7.3) potassium channels, ultimately helping to control the occurrence of epilepsy. Additionally, KCNQ channel mRNA and protein can also be expressed in oligodendrocytes, microglia, and astrocytes (39). Although glial cells are not excitable in the same way as neurons, they do express other voltage-gated ion channels and can experience transient changes in membrane potential and intracellular calcium levels (40). This suggests that glial cells may dynamically regulate neuronal function. Specifically, the KCNQ channels in glial cells mediate the release of GABA by regulating the function of L-type voltage-gated calcium channels, thereby influencing neuronal excitability (41).

It is therefore not difficult to infer that calcium channels are closely related to seizures. Voltage-gated calcium (Cav) channels are found at the presynaptic endings, dendrites, and axon initial segments (AIS) of pyramidal and intermediate neurons (42). These channels are subdivided into three main families: Cav1 (L-type), Cav2 (N-type, P/Q-type, and R-type), and Cav3 (T-type and R-type). Mutations in all three families of calcium channels have been implicated in epilepsy (43). Recently, particular attention has been paid to the role of the T-type Cav3.2 channel in epilepsy (44). Excessive opening of Cav3.2 channels leads to an increase in low-threshold calcium current, which promotes depolarization and explosive neuronal discharge (45). In contrast, Cav3.2 knockout mice show a reduction in absence seizures, highlighting the critical role of this channel in seizure activity (46). Due to the correlation between calcium and potassium channels, gene therapy approaches targeting the overexpression of calcium-activated potassium channels are continuously being optimized. The advantage of selecting this subgroup of channels for gene therapy lies in the ability to maximize the activation of calcium-activated potassium channels and their hyperpolarization effects during intracellular calcium accumulation (47). This process can be accurately monitored during epileptic activities in neural networks, providing a precise means to regulate neuronal excitability and potentially mitigate seizure events.

The mechanism of transporter effects, particularly that of glutamate transporters, is a current hot research topic. Glutamate, the primary excitatory neurotransmitter in the mammalian central nervous system, plays a crucial role in normal brain function (48). It is stored in synaptic vesicles within presynaptic terminals, which fuse with the plasma membrane in response to an increase in action potential and intracellular calcium ions (22). This fusion releases glutamate into the synaptic cleft, where it activates receptors on the postsynaptic neurons. This activation triggers action potentials in the postsynaptic neurons and further propagates signal conduction under physiological conditions.

The excitatory amino acid transporter (EAAT) is a secondary active transporter that couples the movement of glutamate with the co-transport of three sodium ions (49), one hydrogen ion, and the reverse transport of one potassium ion. EAAT2, the most abundant subtype (50), is widely expressed in the brain and spinal cord, making it a key target in research. When EAAT2 expression is epigenetically inhibited, extracellular glutamate concentrations accumulate, leading to excitotoxic damage and the development of epilepsy (51).

As discussed above, it is clear that the molecular mechanisms of ion channels are largely related to *γ*-aminobutyric acid (GABA), the most important inhibitory neurotransmitter in the brain, which plays a critical role in the onset and progression of epilepsy (52). Abnormalities in GABA metabolism, including disturbances in GABA synthesis, transport, gene encoding of the GABA receptor, and GABA inactivation, can lead to epilepsy (53).

The dynamic reversal mechanism of excitatory and inhibitory neurotransmitters is also a major area of focus in current research. The GABAA receptor, a ligand-gated chloride channel, mediates rapid synaptic inhibition. When GABA binds to the GABAA receptor, the ion channels open, allowing chloride ions to flow in or out of the cell (54). The direction of chloride ion flow is determined by the concentration gradient and is regulated by KCC2 and NKCC1. The developmental expression patterns of NKCC1 and KCC2 are dynamic, as the expression of KCC2 increases during development. The ratio of KCC2 to NKCC1 rises over time, stabilizing around the age of 2 (55). This shift is considered crucial for the transformation of GABAergic signaling from an excitatory to an inhibitory role, marking an important transition in neuronal function and network stability. In immature neurons of the embryonic nervous system, NKCC1 predominates, leading to an increase in intracellular chloride concentration. This results (6) in GABA-mediated chloride efflux and depolarization of the cell membrane, which enhances neuronal excitability. This process is crucial for the excitability, differentiation, migration, and proliferation of neurons. In mature neurons, KCC2 dominates, lowering the intracellular chloride concentration and causing GABA-mediated chloride influx, which hyperpolarizes the cell membrane and inhibits neuronal activity.

The proper expression of each subunit gene of the GABAA receptor is essential for its function. It is the balance between excitation and inhibition that maintains the stability of the nervous system. However, when mutations on one side of this balance occur, it can lead to neurological disorders, such as epilepsy. As a potential mechanism of epileptic seizures (56), ion channel dysfunction relies on long-term reversibility (lasting months rather than hours), making the continuous monitoring of EEG (cEEG) necessary. Simultaneously, it is essential to consider the aggregation of seizures and intervene proactively using drugs or other therapeutic techniques. The inherent unpredictability of epileptic seizures has led to a diversification of research, with many emerging technologies being explored, such as optogenetic methods (57) and the integration of artificial intelligence (58) for seizure prediction and intervention. In Figure 6A, the presence of keywords such as “identification” and “model” suggests that current research may be increasingly incorporating AI-based recognition and big data modeling, aligning with the broader context of rapid technological advancement. However, Anti-Seizure Medications (ASMs) remain the cornerstone of epilepsy management (59). Based on this, the study summarizes the pathogenesis discussed and the corresponding drug treatments, as outlined in Table 3.

**Table 3:** The genes in the article and related diseases and treatments of them.

Gene	Related disease	Treatment
SCN1A (69)	Dravet syndrome, Genetic epilepsy with febrile seizures plus(GEFS+)	Valproate, Clobazam, avoid using sodium channel blockers
SCN2A (24, 70)	West syndrome, Early onset epileptic encephalopathy(EOEE)	Sodium vedproate, phenobarbital, sodium channel blockers like carbamazepine
SCN3A (42, 71)	Early onset epileptic encephalopathy(EOEE), Focal epilepsy	Similar treatments to SCN2A
SCN8A (24, 72)	Early onset epileptic encephalopathy(EOEE), Benign Familial Neonatal Seizures(BFNIE)	Similar treatments to SCN2A
KCNQ2 (73–75)	Benign Familial Neonatal Epilepsy (BFNE), Encephalopathy	Carbamazepine, KCNQ openers like cannabidiol
KCNQ3 (73–75)	Benign Familial Neonatal Epilepsy (BFNE), Developmental Delay	Similar treatments to KCNQ2, selective channel openers

### 4.2 Future hotspot prediction of epilepsy

Both the literature clustering (Figure 7A) and keyword clustering (Figure 6B) reveal the emergence of the keyword “autoimmune encephalitis,” which reflects the increasing focus on the neuroimmune interaction mechanism in current research. Specifically, this involves (60) exploring the role of microglia-mediated synaptic pruning in the development and progression of epilepsy. As mentioned in previous articles, the structural and functional breakdown of the balance between excitation (E) and inhibition (I) synapses, known as the synaptic E/I balance, is a key factor in the development of various central nervous system diseases (61). While most studies on epilepsy focus on abnormally high excitability, the complexity of its pathogenesis and the diversity of research directions have created a strong desire to identify new research targets. Microglia, as resident immune cells in the central nervous system (62), play a crucial role in maintaining homeostasis and have become a focal point due to the interaction between epilepsy and neuroinflammation. One of the important functions of microglia is synaptic pruning (61), which involves receiving information from the intestinal microbiota via the vagus nerve connection. This process positions microglia as a potential hub that could be involved in triggering epileptic seizures (62). However, despite its relatively recent emergence, microglia research holds high expectations in the field, particularly due to its potential when combined with various research methodologies.

In Figure 7, the #exome category also warrants attention, aligning with keywords such as “genes.” With the continuous advancement of science and technology, next-generation sequencing (NGS) has become a reliable diagnostic tool for epilepsy treatment (63). The genetic mechanisms underlying epilepsy are complex, involving copy number variations (CNV), single nucleotide variations (SNV), small insertions or deletions (indels), and dynamic variations (64). There is a significant phenotypic overlap among variations in various epilepsy-related genes. The widespread adoption of NGS has greatly enhanced the molecular diagnosis of epilepsy, enabling the rapid identification of pathogenic genes, which in turn supports prognosis evaluation and the implementation of precision medicine (65). Optogenetics, which combines optics and genetics, has become a key focus in preclinical research (66). By stimulating specific epileptic targets using optogenetics, researchers can trigger epileptic activity and observe the spread patterns of seizures in real-time (67). However, despite its promise, there remain substantial challenges in translating optogenetic techniques into clinical practice. The nano-delivery system faces similar challenges. While it holds great promise in the design of epilepsy treatments, its successful implementation in clinical practice still requires resolution of various obstacles (68). As mentioned repeatedly, due to the diversity of epilepsy pathogenesis, there remains a significant gap in the research landscape in this field. However, with the continued progress and development of science and technology, there is great anticipation for more breakthroughs and results that will further our understanding and treatment of epilepsy.

## Data Availability

The original contributions presented in the study are included in the article/Supplementary material, further inquiries can be directed to the corresponding author.

## Glossary

E: Excitation
I: Inhibition
Kv: Voltage-gated potassium
Nav: Voltage-gated sodium
Cav: Voltage-gated calcium
DBS: Deep brain stimulation
RNS: Repeated nerve stimulation
AAv: Adeno-associated virus
FCD: Focal cortical dysplasia
GOF: Gain-of-function
AIS: Axon initial segments
EEG: Electroencephalogram
NGS: Next-generation sequencing
CNV: Copy number variations
SNV: Single nucleotide variations
GABA: *γ*-aminobutyric acid
EAAT: Excitatory amino acid transporter
KCC2: K + -Cl- cotransporter 2
NKCC: Na + -K + -2Cl- cotransporter
ASMs: Anti-Seizure Medications
KCNQ2: Potassium voltage-gated channel, member 2
KCNQ3: Potassium voltage-gated channel, member 3
SCN1A: Sodium Voltage-Gated Channel Alpha Subunit 1
SCN2A: Sodium Voltage-Gated Channel Alpha Subunit 2
SCN3A: Sodium Voltage-Gated Channel Alpha Subunit 3
SCN8A: Sodium Voltage-Gated Channel Alpha Subunit 8
4-PBA: 4-Phenylbutyric acid
SUDEP: Sudden Unexplained Death in Epilepsy

## References

[1] Zhong DL Luo SX Zheng LL Zhang YG Jin RJ. Epilepsy occurrence and circadian rhythm: a bibliometrics study and visualization analysis via CiteSpace. Front Neurol. (2020) 11. doi: 10.3389/fneur.2020.00984, PMID: 33250835 PMC7674827

[2] Cihan E Devinsky O Hesdorffer DC Brandsoy M Li L Fowler DR . Temporal trends and autopsy findings of SUDEP based on medico-legal investigations in the United States. Neurology. (2020) 95:E867. doi: 10.1212/wnl.0000000000009996, PMID: 32636323 PMC7605498

[3] Guo Y Xu ZYR Cai MT Gong WX Shen CH. Epilepsy with suicide: a bibliometrics study and visualization analysis via CiteSpace. Front Neurol. (2022) 12:474. doi: 10.3389/fneur.2021.823474, PMID: 35111131 PMC8802777

[4] Reid CA. Preface: special issue: "ion channels and genetic epilepsy". J Neurochem. (2024) 168:3829–30. doi: 10.1111/jnc.16121, PMID: 38722169

[5] Chen B Xu CL Wang Y Lin WK Wang Y Chen LY . A disinhibitory nigra-parafascicular pathway amplifies seizure in temporal lobe epilepsy. Nat Commun. (2020) 11:648. doi: 10.1038/s41467-020-14648-8, PMID: 32066723 PMC7026152

[6] Hui KK Chater TE Goda Y Tanaka M. How staying negative is good for the (adult) brain: maintaining chloride homeostasis and the GABA-shift in neurological disorders. Front Mol Neurosci. (2022) 15:111. doi: 10.3389/fnmol.2022.893111, PMID: 35875665 PMC9305173

[7] Rubio C Romo-Parra H López-Landa A Rubio-Osornio M. Classification of current experimental models of epilepsy. Brain Sci. (2024) 14:1024. doi: 10.3390/brainsci14101024, PMID: 39452036 PMC11506208

[8] Hodges SL Lugo JN. Therapeutic role of targeting mTOR signaling and neuroinflammation in epilepsy. Epilepsy Res. (2020) 161:106282. doi: 10.1016/j.eplepsyres.2020.106282, PMID: 32036255 PMC9205332

[9] Zummo L Vitale AM Bavisotto CC De Curtis M Garbelli R Giallonardo AT . Molecular chaperones and miRNAs in epilepsy: pathogenic implications and therapeutic prospects. Int J Mol Sci. (2021) 22:601. doi: 10.3390/ijms22168601, PMID: 34445306 PMC8395327

[10] Kim HK Gschwind T Nguyen TM Bui AD Felong S Ampig K . Optogenetic intervention of seizures improves spatial memory in a mouse model of chronic temporal lobe epilepsy. Epilepsia. (2020) 61:561–71. doi: 10.1111/epi.16445, PMID: 32072628 PMC7708390

[11] Pale U Teijeiro T Rheims S Ryvlin P Atienza D. Combining general and personal models for epilepsy detection with hyperdimensional computing. Artif Intell Med. (2024) 148:102754. doi: 10.1016/j.artmed.2023.102754, PMID: 38325932

[12] Huang YR Wang QH Liu XY Du WJ Hao ZJ Wang YW. Transcriptional signatures of a dynamic epilepsy process reveal potential immune regulation. Mol Neurobiol. (2024) 61:3384–96. doi: 10.1007/s12035-023-03786-x, PMID: 37989981 PMC11087345

[13] Zoungrana LI Didik S Wang H Slotabec L Li J. Activated protein C in epilepsy pathophysiology. Front Neurosci. (2023) 17:17. doi: 10.3389/fnins.2023.1251017, PMID: 37901428 PMC10603301

[14] Melin E Andersson M Gotzsche CR Wickham J Huang YZ Szczygiel JA . Combinatorial gene therapy for epilepsy: gene sequence positioning and AAV serotype influence expression and inhibitory effect on seizures. Gene Ther. (2023) 30:649–58. doi: 10.1038/s41434-023-00399-w 37029201 PMC10457185

[15] Senel E Demir E. Bibliometric analysis of apitherapy in complementary medicine literature between 1980 and 2016. Complement Ther Clin Pract. (2018) 31:47–52. doi: 10.1016/j.ctcp.2018.02.003, PMID: 29705479

[16] Sun J Guo Y Scarlat MM Lv G Yang XG Hu YC. Bibliometric study of the orthopaedic publications from China. Int Orthop. (2018) 42:461–8. doi: 10.1007/s00264-018-3828-8, PMID: 29464369

[17] Shorvon S Guerrini R. Acute symptomatic seizures-should we retain the term? Epilepsia. (2010) 51:722–3. doi: 10.1111/j.1528-1167.2010.02501.x, PMID: 20394648

[18] Ramirez-Franco J Debreux K Sangiardi M Belghazi M Kim Y Lee SH . The downregulation of Kv1 channels in Lgi1−/− mice is accompanied by a profound modification of its interactome and a parallel decrease in Kv2 channels. Neurobiol Dis. (2024) 196:106513. doi: 10.1016/j.nbd.2024.106513, PMID: 38663634

[19] Chow CY Chin YKY Ma LL Undheim EAB Herzig V King GF. A selective NaV1.1 activator with potential for treatment of Dravet syndrome epilepsy. Biochem Pharmacol. (2020):181. doi: 10.1016/j.bcp.2020.113991 32335140

[20] Ma DM Zheng YM Li XX Zhou XY Yang ZN Zhang Y . Ligand activation mechanisms of human KCNQ2 channel. Nat Commun. (2023) 14:416. doi: 10.1038/s41467-023-42416-x, PMID: 37857637 PMC10587151

[21] Huang J Fan X Jin XQ Lyu C Guo QM Liu T . Structural basis for human Cav3.2 inhibition by selective antagonists. Cell Res. (2024) 34:440–50. doi: 10.1038/s41422-024-00959-8, PMID: 38605177 PMC11143251

[22] Green JL dos Santos WF Fontana ACK. Role of glutamate excitotoxicity and glutamate transporter EAAT2 in epilepsy: opportunities for novel therapeutics development. Biochem Pharmacol. (2021) 193:114786. doi: 10.1016/j.bcp.2021.114786, PMID: 34571003 PMC8605998

[23] Scheffer IE Berkovic S Capovilla G Connolly MB French J Guilhoto L . ILAE classification of the epilepsies: position paper of the ILAE commission for classification and terminology. Epilepsia. (2017) 58:512–21. doi: 10.1111/epi.13709, PMID: 28276062 PMC5386840

[24] Ademuwagun IA Rotimi SO Syrbe S Ajamma YU Adebiyi E. Voltage gated sodium channel genes in epilepsy: mutations, functional studies, and treatment dimensions. Front Neurol. (2021) 12:50. doi: 10.3389/fneur.2021.600050, PMID: 33841294 PMC8024648

[25] Martins Custodio H Clayton LM Bellampalli R Pagni S Silvennoinen K Caswell R . Widespread genomic influences on phenotype in Dravet syndrome, a 'monogenic' condition. Brain. (2023) 46:3885–97. doi: 10.1093/brain/awad111 PMC10473570 37006128

[26] Ma R Duan YR Zhang LP Qi XH Zhang L Pan SP . SCN1A-related epilepsy: novel mutations and rare phenotypes. Front Mol Neurosci. (2022) 15:183. doi: 10.3389/fnmol.2022.826183, PMID: 35663268 PMC9162153

[27] Tuchman R. Autism and cognition within epilepsy: social matters. Epilepsy Curr. (2015) 15:202–5. doi: 10.5698/1535-7511-15.4.202, PMID: 26316868 PMC4532233

[28] Yamagata T Raveau M Kobayashi K Miyamoto H Tatsukawa T Ogiwara I . CRISPR/dCas9-based Scn1a gene activation in inhibitory neurons ameliorates epileptic and behavioral phenotypes of Dravet syndrome model mice. Neurobiol Dis. (2020) 141:104954. doi: 10.1016/j.nbd.2020.104954, PMID: 32445790

[29] Yuan YK Lopez-Santiago L Denomme N Chen CL O'Malley HA Hodges SL . Antisense oligonucleotides restore excitability, GABA signalling and sodium current density in a Dravet syndrome model. Brain. (2024) 147:1231–46. doi: 10.1093/brain/awad349, PMID: 37812817 PMC10994531

[30] Doganyigit Z Okan A Akyüz E Yilmaz S Ates S Taheri S . Can endoplasmic reticulum stress observed in the PTZ-kindling model seizures be prevented with TUDCA and 4-PBA? Eur J Pharmacol. (2023) 960:176072. doi: 10.1016/j.ejphar.2023.176072, PMID: 37852571

[31] Lee S Kim SH Kim B Lee ST Choi JR Kim HD . Genetic diagnosis and clinical characteristics by etiological classification in early-onset epileptic encephalopathy with burst suppression pattern. Epilepsy Res. (2020) 163:323. doi: 10.1016/j.eplepsyres.2020.106323, PMID: 32247221

[32] Skjei KL Church EW Harding BN Santi M Holland-Bouley KD Clancy RR . Clinical and histopathological outcomes in patients with SCN1A mutations undergoing surgery for epilepsy. J Neurosurg Pediatr. (2015) 16:668–74. doi: 10.3171/2015.5.Peds14551, PMID: 26339958

[33] Kiani L. Gene therapy for seizures in focal cortical dysplasia. Nat Rev Neurol. (2024) 20:63–3. doi: 10.1038/s41582-023-00926-8, PMID: 38167679

[34] Zheng YT Chen J. Voltage-gated potassium channels and genetic epilepsy. Front Neurol. (2024) 15:75. doi: 10.3389/fneur.2024.1466075, PMID: 39434833 PMC11492950

[35] Nappi P Miceli F Soldovieri MV Ambrosino P Barrese V Taglialatela M. Epileptic channelopathies caused by neuronal Kv7 (KCNQ) channel dysfunction. Pflugers Arch. (2020) 472:881–98. doi: 10.1007/s00424-020-02404-2, PMID: 32506321

[36] Nappi M Barrese V Carotenuto L Lesca G Labalme A Ville D . Gain of function due to increased opening probability by two KCNQ5 pore variants causing developmental and epileptic encephalopathy. Proc Natl Acad Sci U S A. (2022) 119:e2116887119. doi: 10.1073/pnas.2116887119, PMID: 35377796 PMC9169635

[37] Alaimo A Etxeberria A Gómez-Posada JC Gomis-Perez C Fernández-Orth J Malo C . Lack of correlation between surface expression and currents in epileptogenic AB-calmodulin binding domain Kv7.2 potassium channel mutants. Channels. (2018) 12:512. doi: 10.1080/19336950.2018.1511512, PMID: 30126342 PMC6161613

[38] Vanoye CG Desai RR Ji ZG Adusumilli S Jairam N Ghabra N . High-throughput evaluation of epilepsy-associated KCNQ2 variants reveals functional and pharmacological heterogeneity. JCI Insight. (2022) 7:314. doi: 10.1172/jci.insight.156314, PMID: 35104249 PMC8983144

[39] Nam MH Cho J Kwon DH Park JY Woo J Lee J . Excessive astrocytic GABA causes cortical hypometabolism and impedes functional recovery after subcortical stroke. Cell Rep. (2020) 32:7861. doi: 10.1016/j.celrep.2020.107861, PMID: 32640227

[40] Chang A Abderemane-Ali F Hura GL Rossen ND Gate RE Minor DL. A Calmodulin C-lobe ca(2+)-dependent switch governs Kv7 channel function. Neuron. (2018) 97:35. doi: 10.1016/j.neuron.2018.01.035, PMID: 29429937 PMC5823783

[41] Weston MC. KCN channels "Cue" up GABA release from astrocytes. Epilepsy Curr. (2024) 24:429–30. doi: 10.1177/15357597241280504, PMID: 39540125 PMC11556643

[42] Debanne D Mylonaki K Musella ML Russier M. Voltage-gated ion channels in epilepsies: circuit dysfunctions and treatments. Trends Pharmacol Sci. (2024) 45:1018–32. doi: 10.1016/j.tips.2024.09.004 39406591

[43] Lauerer RJ Lerche H. Voltage-gated calcium channels in genetic epilepsies. J Neurochem. (2024) 168:3853–71. doi: 10.1111/jnc.15983, PMID: 37822150 PMC11591408

[44] Harding EK Dedek A Bonin RP Salter MW Snutch TP Hildebrand ME. The t-type calcium channel antagonist, Z944, reduces spinal excitability and pain hypersensitivity. Br J Pharmacol. (2021) 178:3517–32. doi: 10.1111/bph.15498, PMID: 33871884 PMC8453510

[45] Petersen AV Jensen CS Crépel V Falkerslev M Perrier JF. Serotonin regulates the firing of principal cells of the subiculum by inhibiting a T-type Ca^2+^ current. Front Cell Neurosci. (2017) 11:60. doi: 10.3389/fncel.2017.00060 28326015 PMC5339341

[46] Casillas-Espinosa PM Lin RX Li R Nandakumar NM Dawson G Braine EL . Effects of the T-type calcium channel CaV3.2 R1584P mutation on absence seizure susceptibility in GAERS and NEC congenic rats models. Neurobiol Dis. (2023) 184:217. doi: 10.1016/j.nbd.2023.106217, PMID: 37391087

[47] Nikitin ES Balaban PM Zaitsev AV. Prospects for gene therapy of epilepsy using calcium-acivated potassium channel vectors. J Evol Biochem Physiol. (2022) 58:1065–74. doi: 10.1134/s0022093022040111

[48] Fontana ACK. Current approaches to enhance glutamate transporter function and expression. J Neurochem. (2015) 134:982–1007. doi: 10.1111/jnc.13200, PMID: 26096891

[49] Vandenberg RJ Ryan RM. Mechanisms of glutamate transport. Physiol Rev. (2013) 93:1621–57. doi: 10.1152/physrev.00007.2013, PMID: 24137018

[50] Tanaka K. Expression cloning of a rat glutamate transporter. Neurosci Res. (1993) 16:149–53. doi: 10.1016/0168-0102(93)90082-2, PMID: 8387171

[51] Sha LZ Li GJ Zhang XN Lin YR Qiu YJ Deng Y . Pharmacological induction of AMFR increases functional EAAT2 oligomer levels and reduces epileptic seizures in mice. JCI Insight. (2022) 7:247. doi: 10.1172/jci.insight.160247, PMID: 35938532 PMC9462477

[52] Sanacora G Mason GF Rothman DL Hyder F Ciarcia JJ Ostroff RB . Increased cortical GABA concentrations in depressed patients receiving ECT. Am J Psychiatry. (2003) 160:577–9. doi: 10.1176/appi.ajp.160.3.577, PMID: 12611844

[53] Feng Y Wei ZH Liu C Li GY Qiao XZ Gan YJ . Genetic variations in GABA metabolism and epilepsy. Seizure. (2022) 101:22–9. doi: 10.1016/j.seizure.2022.07.007, PMID: 35850019

[54] Hannan S Minere M Harris J Izquierdo P Thomas P Tench B . GABAAR isoform and subunit structural motifs determine synaptic and extrasynaptic receptor localisation. Neuropharmacology. (2020) 169:107540. doi: 10.1016/j.neuropharm.2019.02.022, PMID: 30794836

[55] Hyde TM Lipska BK Ali T Mathew SV Law AJ Metitiri OE . Expression of GABA signaling molecules KCC2, NKCC1, and GAD1 in cortical development and schizophrenia. J Neurosci. (2011) 31:11088–95. doi: 10.1523/jneurosci.1234-11.2011, PMID: 21795557 PMC3758549

[56] Kipnis PA Kadam SD. Novel concepts for the role of chloride cotransporters in refractory seizures. Aging Dis. (2021) 12:1056–69. doi: 10.14336/ad.2021.0129, PMID: 34221549 PMC8219493

[57] Wang Y Xu CL Xu ZH Ji CH Liang J Wang Y . Depolarized GABAergic signaling in subicular microcircuits mediates generalized seizure in temporal lobe epilepsy. Neuron. (2017) 1:92–5. doi: 10.1016/j.neuron.2017.06.004 28648501

[58] Huo Q Luo X Xu ZC Yang XY. Machine learning applied to epilepsy: bibliometric and visual analysis from 2004 to 2023. Front Neurol. (2024) 15:443. doi: 10.3389/fneur.2024.1374443, PMID: 38628694 PMC11018949

[59] Ghosh S Sinha JK Khan T Devaraju KS Singh P Vaibhav K . Pharmacological and therapeutic approaches in the treatment of epilepsy. Biomedicines. (2021) 9:470. doi: 10.3390/biomedicines9050470, PMID: 33923061 PMC8146518

[60] Wu Q Wang H Liu XY Zhao YJ Su P. Microglial activation and over pruning involved in developmental epilepsy. J Neuropathol Exp Neurol. (2023) 82:150–9. doi: 10.1093/jnen/nlac111, PMID: 36453895

[61] Andoh M Ikegaya Y Koyama R. Synaptic pruning by microglia in epilepsy. J Clin Med. (2019) 8:170. doi: 10.3390/jcm8122170, PMID: 31818018 PMC6947403

[62] Zhang SY Yang XT Wang YP. Bibliometric analysis of the interplay between epilepsy and microglia: trends, hotspots, and emerging research areas. Front Neurol. (2024) 15:823. doi: 10.3389/fneur.2024.1439823, PMID: 39445198 PMC11496296

[63] Gavaz M Aslan ES Tekes S. Clinical application of whole-exome sequencing analysis in childhood epilepsy. J Neurogenet. (2024) 38:187–94. doi: 10.1080/01677063.2024.2434869 39654149

[64] Zou HF Zhang Q Liao JX Zou DF Hu ZQ Li B . Diagnostic efficiency of exome-based sequencing in pediatric patients with epilepsy. Front Genet. (2025) 15:411. doi: 10.3389/fgene.2024.1496411, PMID: 39906551 PMC11790612

[65] Cokyaman T Özcan EG Akbas NE. High genetic diagnostic yield of whole exome sequencing in children with epilepsy and neurodevelopmental disorders. Fetal Pediatr Pathol. (2025) 44:25–39. doi: 10.1080/15513815.2024.2434919 39648350

[66] Ghosh S Sinha JK Ghosh S Sharma H Bhaskar R Narayanan KB. A comprehensive review of emerging trends and innovative therapies in epilepsy management. Brain Sci. (2023) 13:1305. doi: 10.3390/brainsci13091305, PMID: 37759906 PMC10527076

[67] Ledri M Andersson M Wickham J Kokaia M. Optogenetics for controlling seizure circuits for translational approaches. Neurobiol Dis. (2023) 184:106234. doi: 10.1016/j.nbd.2023.106234, PMID: 37479090

[68] Movahedpour A Taghvaeefar R Asadi-Pooya AA Karami Y Tavasolian R Khatami SH . Nano-delivery systems as a promising therapeutic potential for epilepsy: current status and future perspectives. CNS Neurosci Ther. (2023) 29:3150–9. doi: 10.1111/cns.14355, PMID: 37452477 PMC10580365

[69] Wirrell EC Laux L Donner E Jette N Knupp K Meskis MA . Optimizing the diagnosis and management of Dravet syndrome: recommendations from a north American consensus panel. Pediatr Neurol. (2017) 68:18–34. doi: 10.1016/j.pediatrneurol.2017.01.025 28284397

[70] Ogiwara I Miyamoto H Tatsukawa T Yamagata T Nakayama T Atapour N . Nav1.2 haplodeficiency in excitatory neurons causes absence-like seizures in mice. Commun Biol. (2018) 1:99. doi: 10.1038/s42003-018-0099-2, PMID: 30175250 PMC6115194

[71] Holland KD Kearney JA Glauser TA Buck G Keddache M Blankston JR . Mutation of sodium channel SCN3A in a patient with cryptogenic pediatric partial epilepsy. Neurosci Lett. (2008) 433:65–70. doi: 10.1016/j.neulet.2007.12.064, PMID: 18242854 PMC2423278

[72] Veeramah KR O'Brien JE Meisler MH Cheng XY Dib-Hajj SD Waxman SG . De novo pathogenic SCN8A mutation identified by whole-genome sequencing of a family quartet affected by infantile epileptic encephalopathy and SUDEP. Am J Hum Genet. (2012) 90:502–10. doi: 10.1016/j.ajhg.2012.01.006, PMID: 22365152 PMC3309181

[73] De Wachter M Millevert C Nicolai J Cats E Kluger G Milh M . Amitriptyline use in individuals with KCNQ2/3 gain-of-function variants: a retrospective cohort study. Epilepsia. (2025) 66:1628–40. doi: 10.1111/epi.18310, PMID: 39962862 PMC12097469

[74] Nissenkorn A Bar L Ben-Bassat A Rothstein L Abdelrahim H Sokol R . Donepezil as a new therapeutic potential in KCNQ2-and KCNQ3-related autism. Front Cell Neurosci. (2024) 18:442. doi: 10.3389/fncel.2024.1380442, PMID: 39175503 PMC11338814

[75] Weckhuysen S George JAL (2022). *KCNQ2- and KCNQ3-associated epilepsy, of elements in genetics in epilepsy*.

