# Supplementary figures and images for: Bibliometric and visualized analysis of current advances and future directions in epilepsy: from molecular basis to therapy
Source: Front Neurol. 2025 Jul 1;16:1593621. doi: 10.3389/fneur.2025.1593621 (PMC12260245; doi:10.3389/fneur.2025.1593621)

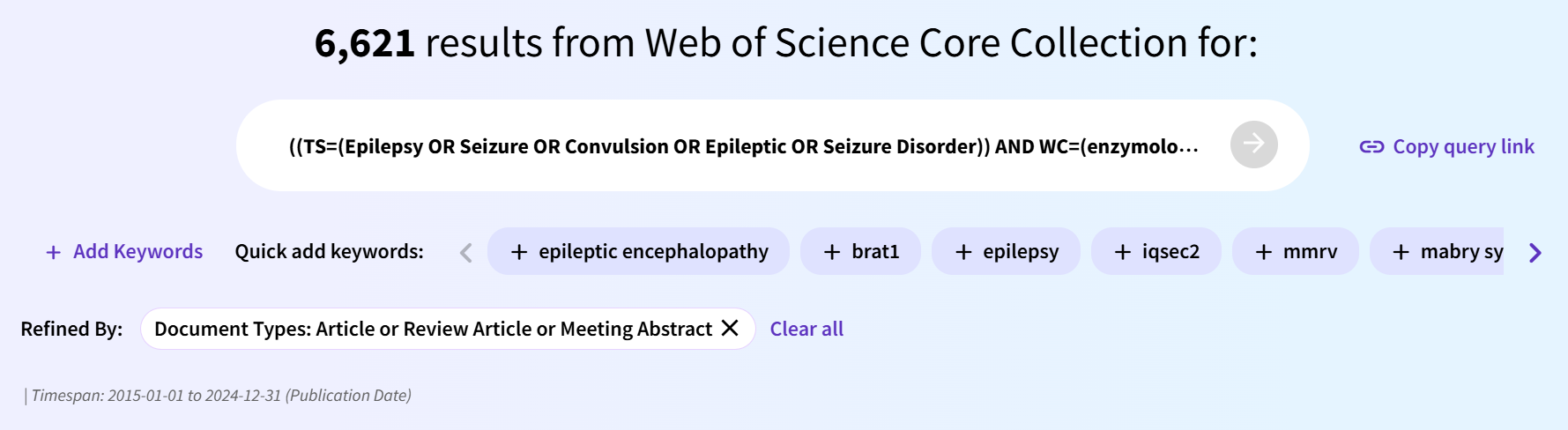

Supplement: Supplementary file 1 [file Image_1.tif]
